# Minimally Invasive Donors Right Hepatectomy versus Open Donors Right Hepatectomy: A Meta-Analysis

**DOI:** 10.3390/jcm12082904

**Published:** 2023-04-17

**Authors:** Chunyang Mu, Chuwen Chen, Jianghong Wan, Guoxin Chen, Jing Hu, Tianfu Wen

**Affiliations:** 1 Department of Liver Surgery, West China Hospital, Sichuan University, Chengdu 610041, China; 2 Department of Outpatient, West China Hospital, Sichuan University, Chengdu 610041, China; 3 Department of Vascular Surgery, West China Hospital, Sichuan University, Chengdu 610041, China; 4 Department of Health Management, West China Fourth Hospital, Sichuan University, Chengdu 610093, China

**Keywords:** liver transplantation, right living donor resection, laparoscopic, laparoscopic-assisted, open living donor resection, meta-analysis

## Abstract

Background: How to obtain a donor liver remains an open issue, especially in the choice of minimally invasive donors right hepatectomy versus open donors right hepatectomy (MIDRH versus ODRH). We conducted a meta-analysis to clarify this question. Methods: A meta-analysis was performed in PubMed, Web of Science, EMBASE, Cochrane Central Register, and ClinicalTrials.gov databases. Baseline characteristics and perioperative outcomes were analyzed. Results: A total of 24 retrospective studies were identified. For MIDRH vs. ODRH, the operative time was longer in the MIDRH group (mean difference [MD] = 30.77 min; *p* = 0.006). MIDRH resulted in significantly less intraoperative blood loss (MD = −57.86 mL; *p* < 0.00001), shorter length of stay (MD = −1.22 days; *p* < 0.00001), lower pulmonary (OR = 0.55; *p* = 0.002) and wound complications (OR = 0.45; *p* = 0.0007), lower overall complications (OR = 0.79; *p* = 0.02), and less self-infused morphine consumption (MD = −0.06 days; 95% CI, −1.16 to −0.05; *p* = 0.03). In the subgroup analysis, similar results were observed in pure laparoscopic donor right hepatectomy (PLDRH) and the propensity score matching group. In addition, there were no significant differences in post-operation liver injury, bile duct complications, Clavien–Dindo ≥ 3 III, readmission, reoperation, and postoperative transfusion between the MIDRH and ODRH groups. Discussion: We concluded that MIDRH is a safe and feasible alternative to ODRH for living donators, especially in the PLDRH group.

## 1. Introduction

Liver transplantation (LT) is an established treatment for patients suffering from end-stage liver disease. Due to a paucity of deceased donors, particularly in Asian countries, living donor liver transplantation (LDLT) has become an important alternative [1]. The LDLT has drawn criticism for the risk it poses to healthy people who will have a major operation without any potential health benefits, including the risk of death. As donor safety is the cornerstone of LDLT, a surgery scheme with less perioperative complication occurrence is crucial.

Open donor liver resection has long been accepted as the classic procedure for obtaining liver for transplant recipients. However, with the conceptualization of minimally invasive liver surgery and the accumulation of laparoscopic techniques, minimally invasive donor left lateral hepatectomy (MIDLH) is considered as standard practice, once the team has fulfilled the adequate learning, because it is minimally invasive and results in less intraoperative blood loss, more rapid postoperative recovery, and a higher level of comfort to patients [2]. Previous physicians had struggled to perform laparoscopic-assisted living donor right hepatectomy, and [3] the first case of pure laparoscopic donor right liver resection (PLDRH) was not reported until 2013 [4] due to the highly complex procedure and intricate anatomy of the human liver. Because surgeons were concerned for the safety of the donor, they were hesitant to employ PLDRH in clinical settings. As a result, the application of minimally invasive donor right hepatectomy (MIDRH) is relatively lagging behind. It should be noted that recent investigations indicated that clinicians prefer MIDRH, in particular, PLDRH, more than open donor right hepatectomy (ODRH) when performing LT [5,6,7]. However, the choice between MIDRH and ODRH remains highly controversial in the liver surgeons’ community.

Therefore, the current meta-analysis was carried out to thoroughly assess the potential advantages of MIDRH over ODRH in LDLT. Our conclusions provide evidence for the selection of clinical strategy that may be advantageous to clinical practitioners as well as patients.

### 1.1. Search Strategy and Study Selection

This study followed the PRISMA guidelines [8]. Published studies which compared MIDRH and ODRH for right liver donor were systematically searched in PubMed, Web of Science, EMBASE, Cochrane Central Register, and ClinicalTrials.gov databases before 30 April 2022, by two independent researchers (CWC, CYM). The combinations of the following key terms were used: laparoscopic, open, conventional, living donor, liver donor, minimally invasive. In order to find additional studies, the references of eligible studies were manually searched.

### 1.2. Inclusion and Exclusion Criteria

Two researchers (JHW, JH) individually screened all titles and abstracts to find papers that qualified: (1) studies focused on comparing MIDRH and ODRH; (2) types of studies that included randomized controlled trials (RCTs), retrospective studies, cohort studies, and case-control studies; (3) articles published in English. The exclusion criteria were as follows: (1) non-English or experimental studies; (2) studies without sufficient data; (3) the publication type was editorials, abstracts, letters, case reports, and expert opinion.

### 1.3. Data Extraction and Quality Assessment

The original data from all candidate articles were independently assessed and extracted by two reviewers (CYM, CWC) by using a unified datasheet which included the following: baseline characteristics (first author, country, publication year, research design, sample size, and mean age, gender, body mass index (BMI), PGV), intraoperative (intraoperative and operative time) and postoperative outcomes (peak AST, peak ALT, peak TB, hospital stay, self-infused morphine consumption, pulmonary complications, bile leak, Clavien–Dindo grade ≥ III, re-hospitalization, reoperation, biliary stricture, postoperative transfusion, wound, postoperative bleeding, and total complications). The Newcastle–Ottawa Scale (NOS) [9] was used to evaluate the quality of included studies and a NOS score ≥ 6 was considered as a high quality article.

### 1.4. Statistical Analysis

Statistical analysis was performed by using Review Manager 5.3 software. The weighted mean difference (WMD) with the 95% confidence interval (CI) and odds ratio (OR) were used to compare continuous variables and dichotomous, respectively. The method of converting medians with ranges into means with standard deviations was in accordance with a prior study carried by Hozo et al. [10] The Higgins I^2^ index was used to quantify the statistical heterogeneity [11]. When heterogeneity is low or moderate (I^2^ < 50%), the fixed-effects model (FEM) was adopted. In contrast, the random-effects model (REM) was adopted when the heterogeneity is high (I^2^ ≥ 50%).

## 2. Results

### 2.1. Search Results and Characteristics of the Included Studies

**Figure 1:**
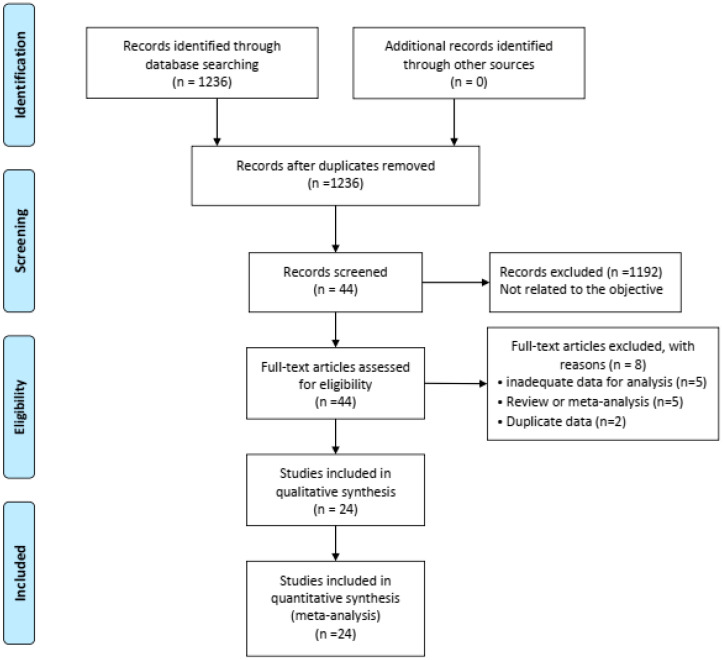
Flowchart of study identification and selection.

**Figure 2:**
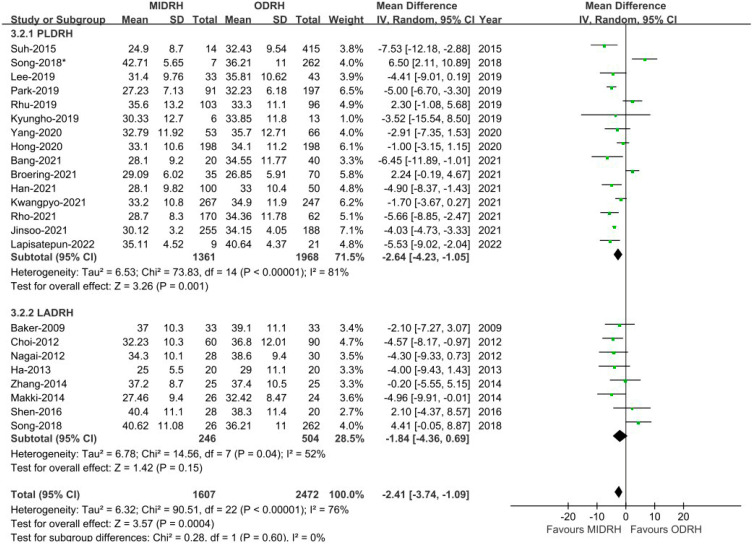
Forest plot of comparison of MIDRH versus ODRH for age [4,5,6,7,12,13,14,15,16,17,19,20,21,22,23,25,26,27,28,29,30,31]. *: Different data in the same article.

**Figure 3:**
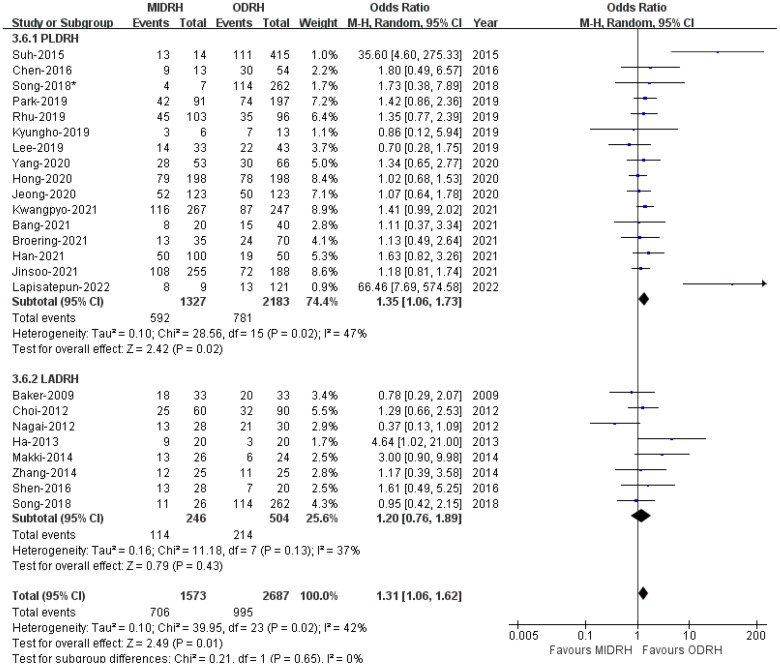
Forest plot of comparison of MIDRH versus ODRH for gender [1,4,5,6,7,12,13,14,15,17,18,19,20,21,22,23,24,25,26,27,29,30,31]. *: Different data in the same article.

Our thorough literature search produced 1236 pertinent English publications in total. Finally, 24 retrospective studies [4,5,6,7,12,13,14,15,16,17,18,19,20,21,22,23,24,25,26,27,28,29,30,31] which involved 4392 patients comparing the MIDRH (1743 patients) and ODRH (2649 patients) were identified for further analysis (Figure 1). According to the different adoptions of laparoscopic techniques, MIDRH was further divided into two groups for categorical subgroup analysis: pure laparoscopic living donor right hepatectomy (PLDRH) and laparoscopic-assisted living donor right hepatectomy (LADRH). The subgroup analysis of studies with propensity score matching encompassed seven studies (out of a total of 1411 patients, 688 and 732 underwent MIDRH and ODRH, respectively). The general information and quality assessment are listed in Table 1. The baseline data showed that the patients who underwent MIDRH were younger (MIDRH vs. ODRH: MD = −2.41; 95% CI, −3.74 to −1.09; *p* = 0.0004, I^2^ = 76%; Table 2, Figure 2) and had a higher ratio of female donors (MIDRH vs. ODRH: 44.9% vs. 37.0%; OR: 1.31; 95% CI: 1.06–1.62; *p* = 0.01; I^2^ = 42%; Table 2, Figure 3). We found that the MIDRH group and the ODRH group were similar with BMI and PGV [4,5,7,12,13,14,15,16,17,18,19,20,21,22,23,24,25,26,27,28,29,30,31] (Table 2).

### 2.2. Perioperative Outcomes

#### Intraoperative Blood Loss

**Figure 4:**
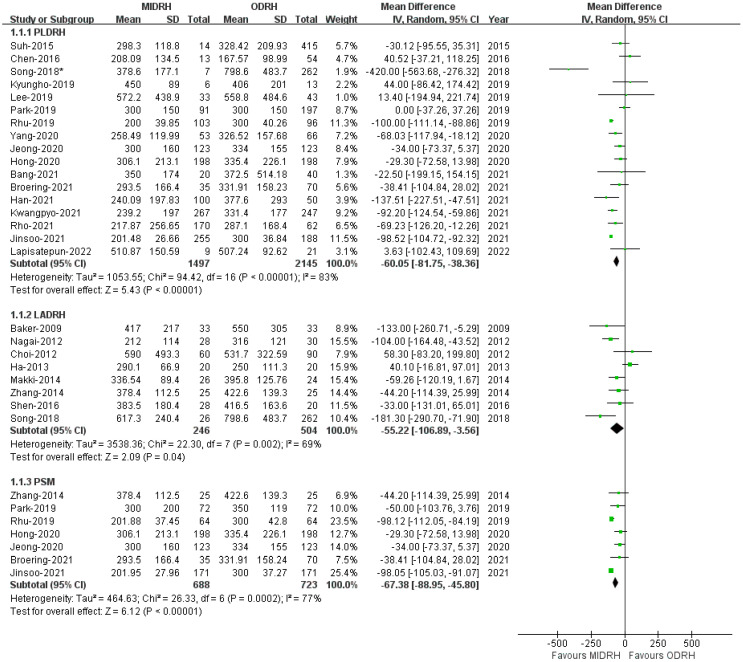
Forest plot comparison of MIDRH versus ODRH for intraoperative blood loss [4,5,6,7,12,13,14,15,16,17,18,19,20,21,22,23,24,25,26,27,28,29,30,31]. *: Different data in the same article.

Intraoperative blood loss was examined by all enrolled studies [4,5,6,7,12,13,14,15,16,17,18,19,20,21,22,23,24,25,26,27,28,29,30,31] (MIDRH 1620 donors vs. ODRH 2649 donors; subgroup: PLDRH 1497 donors vs. ODRH 2145 donors; LADRH 246 donors vs. ODRH 504 donors, respectively). The pooled estimates indicated that the MIDRH group experienced less intraoperative blood loss than the ODRH group (MD = −57.86; 95% CI, −77.58 to −38.1; *p* < 0.00001, I^2^ = 81%, Table 3). Similarly, the intraoperative blood loss experienced by patients who received PLDR [5,6,17,18,20,21,22,23,24,25,26,27,28,29,30,31,32] and LADRH [7,12,13,14,15,16,19,20] was also lower than that experienced by those who received ODRH (PLDRH: MD = −60.05; 95% CI, −81.75 to −38.36; *p* < 0.00001,I^2^ = 83%; LADRH: MD = −55.22; 95% CI, −106.89 to −3.56; *p* = 0.04, I^2^ = 69%, Figure 4). Moreover, our results revealed that patients who received MIDRH had reduced intraoperative blood loss compared to those who underwent ODRH (MD = −67.38; 95% CI, −88.95 to −45.80; *p* < 0.00001, I^2^ = 77%, Figure 4) in the PSM subgroup [16,22,24,25,29,30,31].

### 2.3. Operative Time

**Figure 5:**
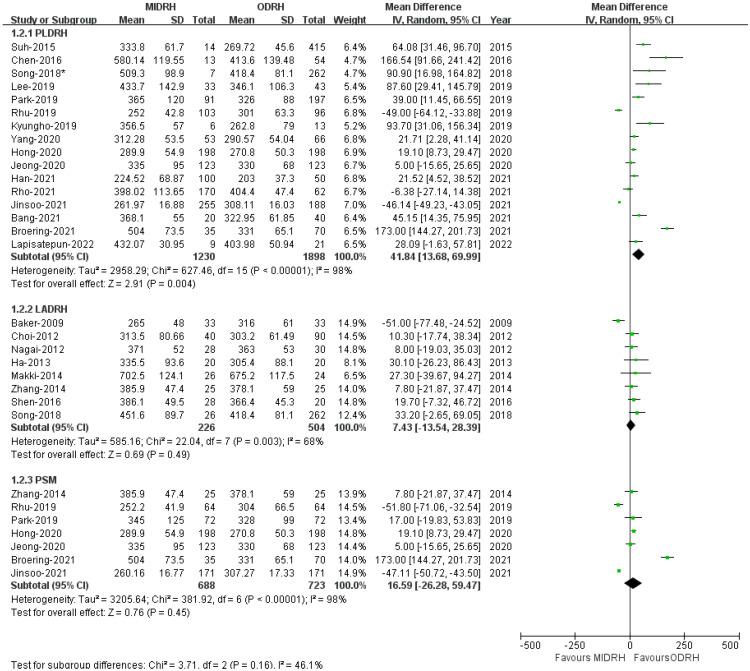
Forest plot comparison of MIDRH versus ODRH for operative time [4,5,7,12,13,14,15,16,17,18,19,20,21,22,23,24,25,26,27,28,29,30,31]. *: Different data in the same article. *: Different data in the same article.

Twenty-three studies [4,5,7,12,13,14,15,16,17,18,19,20,21,22,23,24,25,26,27,28,29,30,31] reported that the length of operation revealed that the MIDRH group’s operating duration was longer than the ORDH group’s (MD = 30.77; 95% CI, 9.03 to 52.15; *p* = 0.006, Table 3), with high heterogeneity observed (I^2^ = 97%). According to the subgroup analysis, the operative time of the PLDRH group [4,5,17,18,20,21,22,23,24,25,26,27,28,29,30,31] was also longer than that of the ODRH group (MD = 41.84; 95% CI, 13.68 to 69.99; *p* = 0.004, I^2^ = 98%, Figure 5). However, the LADRH group [7,12,13,14,15,16,17,18,19,20] and the ODRH group did not differ from one another (MD = 7.43; 95% CI, −13.54 to 28.39; *p* = 0.49, I^2^ = 68%, Figure 5). Besides, the pooled data of the PSM subgroup [16,22,24,25,29,30,31] encompassing 1411 patients suggested that there was no difference in operative time between the MIDRH group and the ODRH group (MD = 16.59; 95% CI, −26.28 to 59.47; *p* = 0.45, I^2^ = 98%, Figure 5).

### 2.4. Length of Hospital Stay (LOS)

**Figure 6:**
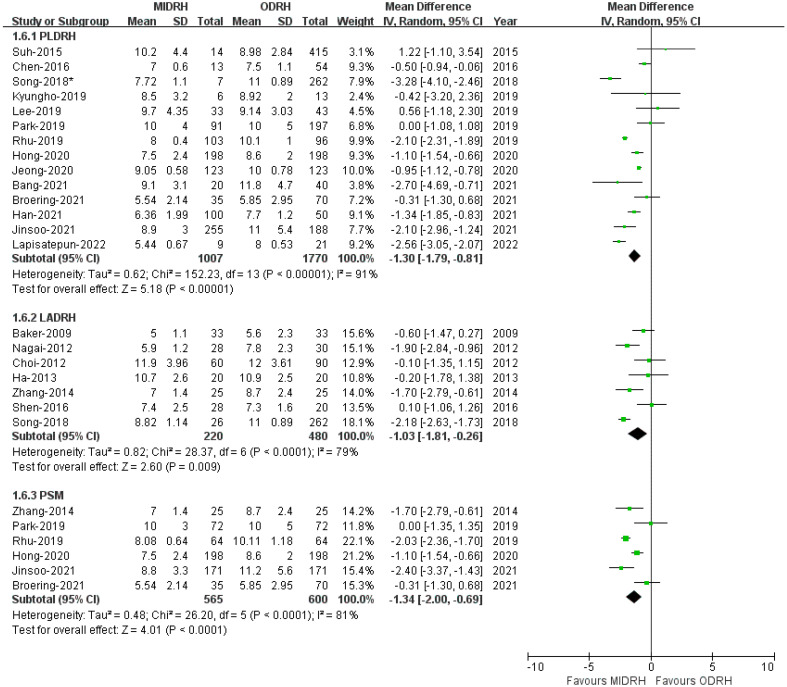
Forest plot and funnel plot comparison of MIDRH versus ODRH for length of hospital stay [4,5,7,12,13,14,16,17,18,19,20,21,22,23,24,25,27,29,30,31]. *: Different data in the same article.

Twenty studies [4,5,7,12,13,14,16,17,18,19,20,21,22,23,24,25,27,29,30,31] revealed that the LOS of patient who underwent MIDRH was shorter than those who underwent ORDH (MD = −1.22; 95% CI, −1.62 to −0.83; *p* < 0.00001, I^2^ = 88%, Table 3). Additionally, categorical subgroup analysis indicated the LOS of the donors in the PLDRH group [4,5,17,18,20,21,22,23,24,25,27,29,30,31] (MD = −1.30; 95% CI, −1.79 to −0.81; *p* < 0.00001, I^2^ = 91%, Figure 6) and the LADRH group [7,12,13,14,16,19,20] (MD = −1.00; 95% CI, −1.81 to −0.26; *p* < 0.0001, I^2^ = 79%, Figure 6) was shorter for both than that of the ORDH group. Furthermore, the PSM subgroup [16,22,25,29,30,31] that included six studies with 1665 patients revealed that the LOS in the MIDRH group was shorter than in the ORDH group (MD = −1.34; 95% CI, −2.00 to −0.69; *p* < 0.0001, I^2^ = 81%, Figure 6).

### 2.5. Pulmonary Complications

**Figure 7:**
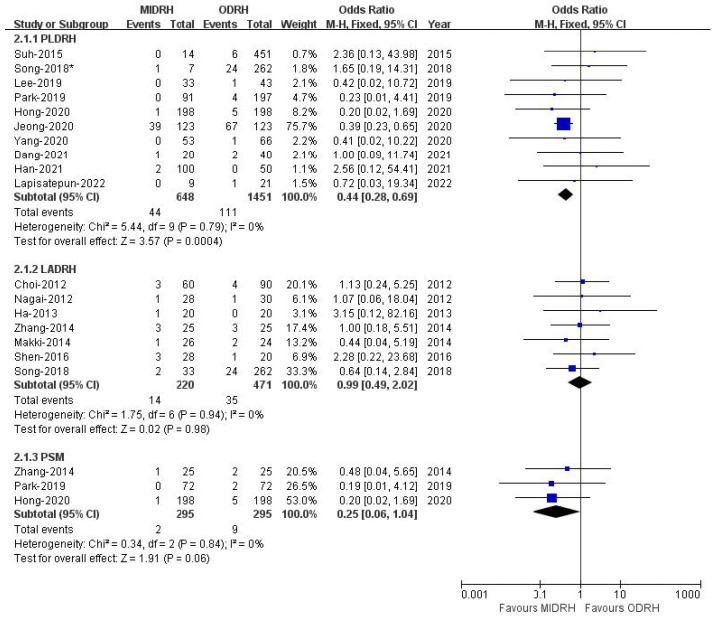
Forest plot comparison of MIDRH versus ODRH for pulmonary complications [4,5,12,13,14,15,16,17,19,20,21,22,24,26,27,30]. *: Different data in the same article.

Pulmonary complications included pleural effusion and pulmonary infection. The pooled data encompassed sixteen studies [4,5,12,13,14,15,16,17,19,20,21,22,24,26,27,30] with a total of 2790 donors and showed that the incidence of pulmonary complications in the MIDRH group was lower than in the ORDH group (OR = 0.55; 95% CI, 0.38 to 0.81; *p* = 0.002, I^2^ = 0%, Table 3). In categorical subgroup analysis, the PLDRH group [4,5,17,20,21,22,24,26,27,30] had a lower pulmonary complication rate (OR = 0.44; 95% CI, 0.28 to 0.69; *p* = 0.0004, I^2^ = 0%, Figure 7). Meanwhile, there was no difference between the LADRH group [12,13,14,15,16,19,20] and the ODRH group (OR = 0.99; 95% CI, 0.49 to 2.02; *p* = 0.98, I^2^ = 0%, Figure 7). Furthermore, the PSM subgroup analysis [16,22,30] suggested that the pulmonary complication rate in the MIDRH group was comparable to that in the ODRH group (OR = 0.25; 95% CI, 0.06 to 1.04; *p* = 0.06, I^2^ = 0%, Figure 7).

### 2.6. Postoperative Transfusion

**Figure 8:**
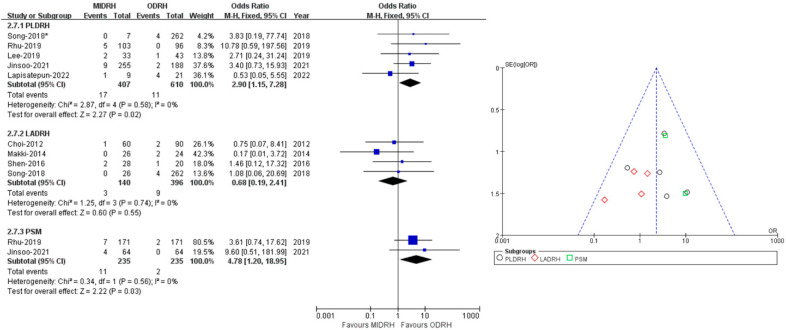
Forest plot comparison of MIDRH versus ODRH for postoperative transfusion [5,12,15,19,20,21,25,29]. *: Different data in the same article.

Eight studies [5,12,15,19,20,21,25,29] encompassing 1553 donors covered the incidence of postoperative transfusion without heterogeneity (I^2^ = 0%). Our results revealed that there was no significant difference between the ODRH group and the MIDRH group (OR = 1.78; 95% CI, 0.88 to 3.59; *p* = 0.11, Table 3) in postoperative transfusion. The LADRH group [12,15,19,20] did not vary from the ODRH group in categorical subgroup analysis, (OR = 0.68; 95% CI, 0.19 to 2.41; *p* = 0.55, I^2^ = 0%, Figure 8). To be noted, the donors in the PLDRH [5,20,21,25,29] group had a lower postoperative transfusion rate than in the ODRH group (OR = 2.90; 95% CI, 1.15 to 7.28; *p* = 0.02, Figure 8). In addition, the PSM subgroup [25,29] analysis, which included two studies, also discovered that the MIDRH group had a lower transfusion rate than the ODRH group (OR = 4.78; 95% CI, 1.20 to 18.95; *p* = 0.03, Figure 8) without heterogeneity (I^2^ = 0%).

### 2.7. Wound

**Figure 9:**
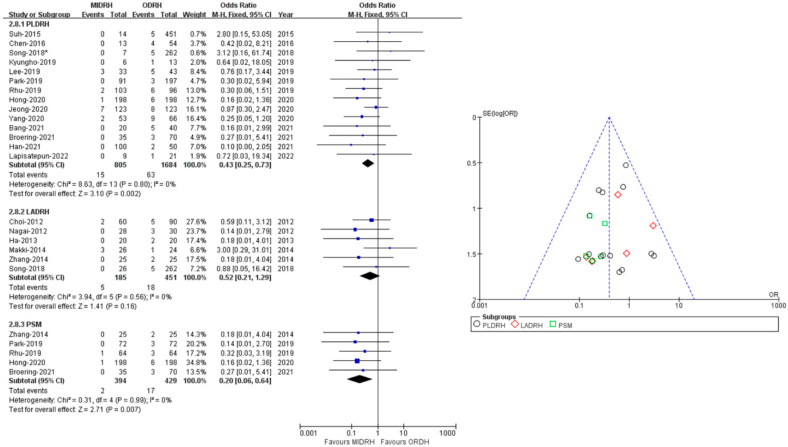
Forest plot and funnel plot comparison of MIDRH versus ODRH for wound complication [4,5,12,13,14,15,16,17,18,20,21,22,23,24,25,26,27,30,31]. *: Different data in the same article.

Nineteen studies [4,5,12,13,14,15,16,17,18,20,21,22,23,24,25,26,27,30,31] including 3125 donors reported wound complications (990 in MIDRH and 2135 in ODRH) without heterogeneity (I^2^ = 0%). It showed that the donors in MIDRH had fewer wound complications than in ODRH (OR = 0.45; 95% CI, 0.29 to 0.71; *p* = 0.0007, Table 3). In addition, in categorical subgroup analysis, the PLDRH group [4,5,17,18,20,21,22,23,24,25,26,27,30,31] also had fewer wound complications than the ODRH group (OR = 0.43; 95% CI, 0.25 to 0.73; *p* = 0.002, Figure 9). Besides, the PSM subgroup [16,22,25,30,31] analysis with pooled data of four studies indicated that the MIDRH group had fewer wound complications than the ODRH group (OR = 0.20; 95% CI, 0.06 to 0.64; *p* = 0.007, Figure 9). However, the LADRH group [12,13,14,15,16,20] had no significant difference from the ODRH group (OR = 0.52; 95% CI, 0.21 to 1.29; *p* = 0.16, Figure 9).

### 2.8. Overall Complication Rate

**Figure 10:**
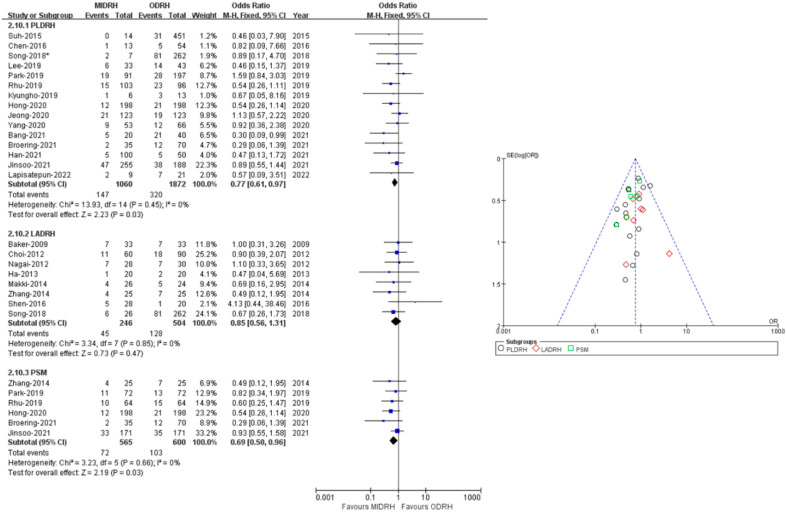
Forest plot and funnel plot comparison of MIDRH versus ODRH for overall complications [4,5,7,12,13,14,15,16,17,18,19,20,21,22,23,24,25,26,27,29,30,31]. *: Different data in the same article.

Twenty-two studies [4,5,7,12,13,14,15,16,17,18,19,20,21,22,23,24,25,26,27,28,29,30] with a total of 3682 donors (MIDRH vs. ODRH: 1306:2376) reported postoperative complications. The pooled data suggested that donors in the MIDRH group had lower incidence of overall complications than the ODRH group (OR = 0.79; 95% CI, 0.64 to 0.96; *p* = 0.02, I^2^ = 0%, Table 3). In categorical subgroup analysis, donors in the PLDRH group [4,17,18,20,21,22,23,24,25,26,27,28,29,30,31] had lower overall complications than in the ODRH group (OR = 0.77; 95% CI, 0.61 to 97; *p* = 0.003, I^2^ = 0%, Figure 10). Additionally, the PSM subgroup [16,22,25,29,30,31] analysis included six studies (MIDRH vs. ODRH: 565:600) and showed that donors had a lower overall complication rate than the ODRH group (OR = 0.69; 95% CI, 0.50 to 0.96; *p* = 0.03, I^2^ = 0%, Figure 10). Meanwhile, there was no difference between the LADRH group [7,12,13,14,15,16,19,20] and the ODRH group (OR = 0.85; 95% CI, 0.56 to 1.31; *p* = 0.47, I^2^ = 0%, Figure 10).

### 2.9. Self-Infused Morphine Consumption (Days)

**Figure 11:**
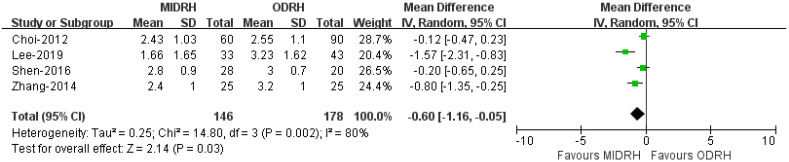
Forest plot and funnel plot comparison of MIDRH versus ODRH for self-infused morphine pump (days) [12,16,19,21].

Four studies [12,16,19,21] including 324 donors reported the use of self-infused morphine consumption. Our results revealed that the donors in the MIDRH group used morphine for fewer days than those in the ODRH group (WMD = −0.06; 95% CI, −1.16 to −0.05; *p* = 0.03, I^2^ = 80%, Figure 11).

### 2.10. Other Outcomes

Our analysis revealed that the MIDRH group and ODRH group were similar with rehospitalization (MIDRH vs. ODRH: 6.5% vs. 3.48%; OR: 1.18; 95% CI: 0.68–2.04; *p* = 0.56, Table 3), reoperation (MIDRH vs ODRH: 3% vs. 1.92%; OR: 1.43; 95% CI: 0.79–2.57; *p* = 0.23, Table 3), Clavien–Dindo grade ≥ III (MIDRH vs. ODRH: 4.8% vs. 4.63%; OR: 1.06; 95% CI: 0.71–1.59; *p* = 0.77, Table 3), peak alanine aminotransferase (MIDRH vs. ODRH: 234.5 ± 112.5 vs. 225.7 ± 135.0; OR: 18.92; 95% CI: −10.26–48.10; *p* = 0.2, Table 3), peak aspartate aminotransferase (MIDRH vs. ODRH: 226.0 ± 104.6 vs. 219.2 ± 121.1; OR: 10.83; 95% CI: −12.57–34.23; *p* = 0.36, Table 3), peak total bilirubin (MIDRH vs. ODRH: 3.1 ± 1.6 vs. 3.1 ± 1.6; OR: −0.08; 95% CI: −0.26–0.09; *p* = 0.36, Table 3), bile leak (MIDRH vs. ODRH: 9.55% vs. 7.48%; OR: 2.57; 95% CI: 0.94–7.00; *p* = 0.07, Table 3), biliary stricture (MIDRH vs. ODRH: 1.55% vs. 0.53%; OR: 2.38; 95% CI: 0.81–7.04; *p* = 0.12, Table 3), and post-operation bleeding (MIDRH vs. ODRH: 1.49% vs. 7.48%; OR: 1.26; 95% CI: 0.59–2.45; *p* = 0.62, Table 3).

### 2.11. Publication Bias

Begg’s funnel plot was drawn for each outcome and adopted to investigate publication bias. All studies lie inside the 95% CI in the funnel plot that indicated no obvious publication bias.

## 3. Discussion

Living donor right hepatectomy is currently the most common donor surgery in adult-to-adult living donor liver transplantation [32,33], in which about two-thirds of the working liver is removed from the donator [34]. Concerns about donor safety and ethical issues have persisted since the procedure was performed in 1996 [34]. Ensuring the safety of the donor is the cornerstone of LDLT. The safety and superiority of minimally invasive hepatectomy have been proved in liver tumor resection [35,36,37,38], and previous studies have also reflected the feasibility and safety of minimally invasive hepatectomy in donor liver resection [39,40,41,42,43]. Moreover, the consensus [2] on minimally invasive donor hepatectomy for living donor liver transplantation stated that “pure laparoscopic” donor hepatectomy is applicable to left lateral hepatectomy and should be considered standard practice once the team has fulfilled the adequate learning. But there is still a lack of high-level evidence to explain the advantages and disadvantages of laparoscopic or open hepatectomy for living right hepatectomy.

In our study, the demographic data showed that the donors who underwent MIDRH were younger and had a female predominance, which was consistent with previous research [44,45,46,47,48,49]. It is easy to understand this phenomenon because the MIDRH has the advantages of quick postoperative recovery, light pain, beautiful appearance, and minimal trauma, and is more favored by the younger and female. Reduced intraoperative blood loss and shorter LOS were found in the MIDRH group, and the average amount of estimated intra-operation blood loss from our pooled data was 283.6 ± 221.8 mL, and 431.4 ± 342.0 mL in the MIDRH and ODRH group. These results were similar to previous studies [44,47,50]. The small amount of estimated intra-operation blood loss may be attributed to the fine dissection, which facilitates the identification and processing of tiny structures. And there were no significant differences in postoperative bleeding and postoperative blood transfusion events between the two groups.

Different from other meta-analyses [44,46,50,51], we found that the procedure time was longer in the MIDRH group than in the ODRH group, especially in PLDRH. However, in the PSM subgroup, the operation time showed no difference. In the encompassing literature, several studies [12,26,29,30] reported a shorter operation time in the PLDRH group than the ODRH group, which included a larger number of cases and is consistent with another study of Lai et al. [52]. This result may be caused by some small sample studies included in our analysis. Due to some limitations of laparoscopic surgery such as motion, visualization, and tactile sensation [53], the learning process for laparoscopy is relatively long. Currently, there are serval reports about the learning curve of PLDRH. Rhu et al. [26] thought that it was possible to reduce the operating time only after more than 50 PLDRH procedures. Lee et al. reported that operating time was stabilized for ODRH after 17 cases and for PLDRH after 15 cases [22]. In our study, there were only two studies with fewer than 15 PLDRH. Meanwhile, the operation time was also affected by the patient’s own conditions. And in cases of tissue structure variation or other anatomical abnormalities, laparoscopy may lead to increased postoperative morbidity [54,55,56]. In general, the operation time of PLDRH will be reduced and the laparoscopic-related complications will be overcome with the accumulation of laparoscopic surgery experience.

Our pooled data indicated that MIDRH had fewer analgesic requirements than ODRH, which was in accordance with the results of previous studies [44,45,46,47,48,49]. MIDRH has a smaller incision without cutting the subcostal nerve and muscle which preserves the integrity of the abdominal wall as much as possible. Regardless of the differences between the operator and the patient, a small incision could promote postoperative rehabilitation, reduce postoperative pain, and improve respiratory status. Meanwhile, our study revealed that MIDRH demonstrated a better surgical incision; this seems to be more evident in PLDRH, with lower wound complication rates. Apparently, it was associated with the hidden benefits of small incision, such as reducing the psychological burden on patients, the rate of infection, and long-term discomfort at the incision site after surgery.

Our study found that the PLDRH group had a favorable advantage in pulmonary complications, which is consistent with previous studies [45,46,51]. This may be associated with the delicate operation being minimally invasive, producing light postoperative pain, and reducing irritation to the chest cavity. Meanwhile, our study found that there was no difference in peak AST, peak ALT, peak TB, bile leak, biliary stricture, Clavien–Dindo grade ≥ III, rehospitalization, and reoperation between MIDRH and ODRH. These indicators had not been investigated in previous studies [46,47,48,49,50,51].

Cost–benefit analysis between MIDRH and ODRH was also important. Riquelme et al. [56] have shown that upfront intraoperative costs associated with ODRH were lower, but the overall costs between ODRH and PLDRH were equivalent after 3 months of follow-up. In our study, data of cost were not reported in the included studies, so we could not conduct a specific analysis on this issue.

There are some limitations in our study. All the articles were retrospective studies without randomized controlled trials. Potential bias exists in the intrinsic retrospective study. Due to time and the fact that times of liver blockage could not be obtained, it was impossible to conduct hierarchical analysis of this research. Some studies had small samples and the outcomes may have been affected by the learning curve. A high level of evidence is still needed to explore the merits of the two surgery procedures.

## 4. Conclusions

In conclusion, MIDRH is a safe and feasible alternative approach in donor right hepatectomy for its better performance in intra-operation blood loss, pulmonary complications, length of stay, postoperative pain, wound complications, and overall complications.

## Figures and Tables

**Table 1:** Characteristics of Included Studies Comparing MIDRH with ODRH for Donors.

Author–Year	Country	Study Type	Study Interval	Sample		Sexy M/F		Age		BMI		PGV		NOS
				MIDRH	ODRH	MIDRH	ODRH	MIDRH	ODRH	MIDRH	ODRH	MIDRH	ODRH	
**LADRH**														
**Baker [7]-2009**	**USA**	**RS**	2004–2007	33	33	15/18	13/20	37 ± 10.3	39.1± 11.1	25.8 ± 4.1	25.9 ± 4.3	900 ± 215	914 ± 160	8
Choi [12]-2012	Korea	RS	2008–2011	60	90	35/25	58/32	32.23 ± 10.3	36.8 ± 12.01	23.33 ± 2.64	23.6 ± 2.94	NA	NA	9
Nagai [13]-2012	USA	RS	2000–2011	28	30	15/13	9/21	34.3 ± 10.1	38.6 ± 9.4	24.0 ± 3.3	30.1 ± 5.1	915 ± 361	800 ± 184	8
Ha [14]-2013	Korea	DCC	2012–2012	20	20	11/9	17/3	25 ± 5.5	29 ± 11.1	23.3 ± 4.0	23.6 ± 3.2	725.1 ± 135.5	755.3 ± 95.7	8
Zhang [16]-2014	China	PSM	2011–2013	25	25	13/12	14/11	37.2 ± 8.7	37.4 ± 10.5	23.8 ± 2.6	22.6 ± 3.0	629.9 ± 128.9	575.2 ± 136.3	9
Makki [15]-2014	India	RS	2011–2013	26	24	13/13	18/6	27.46 ± 9.40	32.42 ± 8.47	24.23 ± 3.64	24.46 ± 4.39	755.50 ± 87.94	725.79 ± 134.35	8
Shen [19]-2016	China	RS	2011–2013	28	20	15/13	13/7	40.4 ± 11.1	38.3 ± 11.4	23.1 ± 1.8	21.9 ± 1.9	634.2 ± 124.2	572.9 ± 122.5	8
Song [20]-2018	China	RS	2001- 2017	26	262	15/11	148/114	40.62± 11.08	36.21 ± 11.00	23.26 ± 2.55	22.95 ± 2.61	NA	NA	8
**PLDRH**														
Suh [17]-2015	Korea	RS	2010–2013	14	415	1/13	304/111	24.9 ± 8.7	32.43 ± 9.54	20.9 ± 2.9	23.08 ± 3.12	NA	NA	8
Chen [18]-2016	China	RS	2013–2015	13	54	4/9	24/30	NA	NA	21.94 ± 2.99	23.08 ± 3.52	605.64 ± 140.47	NA	8
Song [20]-2018 *	China	RS	2001–2017	7	262	3/4	148/114	42.71 ± 5.65	36.21 ± 11.00	23.50 ± 3.23	22.95 ± 2.61	NA	NA	7
Kyungho [23]-2019	Korea	RS	2014–2016	6	13	3/3	6/7	30.33 ± 12.7	33.85 ± 11.8	23.1 ± 2.8	23.5 ± 3.4	NA	NA	8
Lee [21]-2019	Korea	RS	2010–2017	33	43	19/14	21/22	31.4 ± 9.76	35.81 ± 10.62	23.97 ± 6.76	23.07 ± 3.00	750.0 ± 194	725.4 ± 158	9
Park [22]-2019	Korea	RS	2013–2017	91	197	49/42	123/74	27.23 ± 7.13	32.23 ± 6.18	22.69 ± 3.52	23.44 ± 3.57	696 ± 153	703 ± 168	9
Rhu [25]-2019	Korea	RS	2014–2018	103	96	58/45	61/35	35.6 ± 13.2	33.3 ± 11.1	23.8 ± 2.9	23.7 ± 3.1	757 ± 171	745 ± 169	9
Hong [30]-2020	Korea	RS, PSM	2010–2018	198	198	119/79	120/78	33.1 ± 10.6	34.1 ± 11.2	23.7 ± 3.4	23.9 ± 3.2	NA	NA	9
Yang [26]-2020	Korea	RS	2016–2017	53	66	25/28	36/30	32.79 ± 11.92	35.70 ± 12.71	23.49 ± 2.79	23.64 ± 2.68	NA	NA	7
Bang [27]-2021	Korea	RS	2015–2017	20	40	12/8	25/15	28.1 ± 9.2	34.55 ± 11.77	23.7 ± 2.7	23.75 ± 2.84	NA	NA	8
Broering [31]-2021	Italy	PSM	2015–2019	35	70	22/13	46/24	29.09 ± 6.02	26.85 ± 5.91	23.4 ± 2.84	24.1 ± 3.20	701 ± 148	701 ± 133	9
Han [4]-2021	Korea	RS	2012–2019	100	50	50/50	31/19	32.86 ± 9.82	33.0 ± 10.4	22.82 ± 2.94	23.4 ± 3.2	674.89 ± 130.12	722.1 ± 142.0	8
Kwangpyo [6]-2021	Korea	RS	2012–2019	267	247	151/116	160/87	33.2 ± 10.8	34.9 ± 11.9	23.7 ± 3.3	23.3 ± 3.3	716.7 ± 140.1	732.9 ± 153.5	8
Rho [28]-2021	Republic of Korea	RS	2016–2019	170	62	NA	NA	34.36 ± 11.78	28.7 ± 8.3	23.03 ± 2.41	22.1 ± 2.4	764.85 ± 136.43	731.3 ± 124.2	8
Jinsoo Rhu [29]-2021	Korea	RS	2014–2019	255	188	147/108	116/72	30.12 ± 3.2	34.15 ± 4.05	23.4 ± 2.8	23.5 ± 3.0	NA	NA	8
Lapisatepun [5]-2022	Thailand	RS	2015–2021	9	21	1/8	8/13	35.11 ± 4.52	40.64 ± 4.37	22.47 ± 1.0	22.02 ± 1.11	701.91 ± 50.56	724.94 ± 55.62	8
Jeong [24]-2020	Korea	RS, PSM	2013–2018	123	123	71/52	73/50	30.24 ± 3.70	31 ± 3.11	NA	NA	NA	NA	9
**PSM**														
Zhang [16]-2014	China	PSM	2011–2013	25	25	13/12	14/11	37.2 ± 8.7	37.4 ± 10.5	23.8 ± 2.6	22.6 ± 3.0	629.9 ± 128.9	575.2 ± 136.3	9
Park [22]-2019	Korea	PSM	2013–2017	72	72	40/32	43/39	28.5 ± 15	29.5 ± 11.5	23.51 ± 2.83	23.36 ± 3.25	695.5 ± 154.5	716.5 ± 177.5	8
Rhu [25]-2019	Korea	PSM	2014–2018	64	64	39/25	38/26	33.6 ± 12.8	34.1 ± 11.4	23.3 ± 3.2	24 ± 3.2	761 ± 125	764 ± 172	8
Hong [30]-2020	Korea	RS, PSM	2010–2018	198	198	119/79	120/78	33.1 ± 10.6	34.1 ± 11.2	23.7 ± 3.4	23.9 ± 3.2	NA	NA	8
Broering [31]-2021	Italy	PSM	2015–2019	35	70	22/13	46/24	29.09 ± 6.02	26.85 ± 5.91	23.4 ± 2.84	24.1 ± 3.20	701 ± 148	701 ± 133	8
Jinsoo Rhu [29]-2021	Korea	PSM	2014–2020	171	171	104/67	105/66	34.12 ± 3.92	34.12 ± 3.92	23.3 ± 2.7	23.4 ± 3.0	NA	NA	8
Jeong [24]-2020	Korea	RS, PSM	2013–2018	123	123	71/52	73/50	30.24 ± 3.70	31 ± 3.11	NA	NA	NA	NA	8

*: Different data in the same article; MIDRH: minimally invasive donors right hepatectomy; ODRH: open donors right hepatectomy; PLDRH: pure laparoscopic living donor right hepatectomy; LADRH: laparoscopic-assisted living donor right hepatectomy; DCC: double-arm case-controlled study; SD: standard deviation; CI: confidence interval; NOS: Newcastle–Ottawa Scale; RS: retrospective study; PSM: propensity score-matching; NA: not available.

**Table 2:** Pooled donors’ preoperative characteristics.

Variables	No. of Studies	No. of Patients ^#^	MIDRH	ODRH	OR, M-H Fixed, 95% CI OR, Fixed, Random, 95% CI MD, Random, 95% CI	*p* Value	I^2^
**Age, years**	23	4079	32.4 ± 9.8	34.0 ± 14.9	−2.41 [−3.74, −1.09]	0.0004	76%
PLDRH vs. ODRH	15	3329	32.1 ± 9.6	33.5 ± 15.6	−2.64 [−4.23, 1.05]	<0.00001	81%
LADRH vs. ODRH	8	750	34.3 ± 10.9	36.3 ± 11.1	−1.84 [−4.36, 0.69]	0.04	76%
**Gender (Female)**	23	4260	706 (44.9%)	995 (37.0%)	1.31 [1.06, 1.62]	0.01	42%
PLDRH vs. ODRH	15	3510	592 (44.6%)	781 (35.8%)	1.35 [1.06, 1.73]	0.02	47%
LADRH vs. ODRH	8	750	114 (46.3%)	214 (42.5%)	1.20 [0.76, 1.89]	0.79	37%
**BMI, kg/m^2^**	23	4146	23.4 ± 3.2	23.4 ± 3.3	−0.14 [−0.53, 0.24]	0.46	64%
PLDRH vs. ODRH	15	3396	23.4 ± 3.2	23.3 ± 3.1	−0.08 [−0.41, 0.25]	0.63	41%
LADRH vs. ODRH	8	750	23.8 ± 3.2	23.7 ± 3.6	−0.38 [−1.58, 0.82]	0.54	81%
**PGV**	15	1973	730.5 ± 166.4	720.8 ± 159.3	−1.47 [−14.37, 11.44]	0.82	30%
PLDRH vs. ODRH	9	1661	723.1 ± 149.3	717.3 ± 152.9	−7.25 [−21.36, 6.87]	0.31	21%
LADRH vs. ODRH	6	312	768.6 ± 231.9	740.3 ± 190.4	27.90 [−3.93, 59.74]	0.09	16%

^#^: in each group; CI: confidence interval; MIDRH: minimally invasive donors right hepatectomy; ODRH: open donors right hepatectomy; PLDRH: pure laparoscopic living donor right hepatectomy; LADRH: laparoscopic-assisted living donor right hepatectomy; BMI: Body Mass Index; PGV: prospecting liver graft volume.

**Table 3:** Comparison of patient outcomes between MIDRH and ODRH groups.

Variables	No. of Studies	No. of Patients ^#^	MIDRH	ODRH	OR, M-H Fixed, 95% CI OR, Fixed, Random, 95% CI MD, Random, 95% CI	*p* Value	I^2^
**Intraoperative blood loss**	24	4329	283.6 ± 221.8	431.4 ± 342.0	−57.86 [−77.58, −38.14]	<0.00001	81%
**Operative time**	23	3858	330.5 ± 116.1	334.6 ± 96.1	30.77 [9.03, 52.51]	0.006	97%
**Length of Hospital stay**	20	3477	8.3 ± 3.0	9.7 ± 3.3	−1.22 [−1.62, −0.83]	<0.00001	89%
**Pulmonary complications**	9	2790	58 (6.7%)	146 (7.6%)	0.55 [0.38, 0.81]	0.002	0%
**Wound**	20	3125	20 (2.0%)	81 (3.8%)	0.45 [0.29, 0.71]	0.0007	0%
**Total complications**	22	3682	192 (14.7%)	448 (18.9%)	0.79 [0.64, 0.96]	0.02	0%
**Postoperative transfusion**	9	1553	20 (2.6%)	20 (1.6%)	1.78 [0.88, 3.59]	0.11	0%
PLDRH vs. ODRH	5	1017	17 (4.2%)	11 (1.8%)	2.90 [1.15, 7.28]	0.02	0%
LADRH vs. ODRH	4	536	3 (2.1%)	9 (2.3%)	0.68 [0.19, 2.41]	0.55	0%
PSM	2	470	11 (4.7%)	2 (0.85%)	4.78 [1.20, 18.95]	0.03	0%
**Bleeding**	13	2404	11 (1.5%)	21 (1.3%)	1.20 [0.59, 2.45]	0.63	0%
PLDRH vs. ODRH	8	1810	5 (0.9%)	17 (1.4%)	0.80 [0.32, 2.00]	0.14	0%
LADRH vs. ODRH	6	594	6 (3.6%)	4 (0.94%)	2.56 [0.73, 9.05]	0.62	0%
**Peak AST**	18	3030	226.0 ± 104.6	219.2 ± 121.1	10.83 [−12.57, 34.23]	0.36	96%
PLDRH vs. ODRH	11	2366	220.4 ± 82.9	211.3 ± 107.8	13.43 [−15.69, 42.56]	0.37	98%
LADRH vs. ODRH	7	664	250.1 ± 166.8	245.0 ± 154.3	0.39 [−26.86, 27.65]	0.98	44%
PSM	5	1060	214.1 ± 80.9	219.2 ± 77.0	−16.97 [−59.34, 25.40]	0.43	98%
**Peak ALT**	18	3050	234.5 ± 112.5	225.7 ± 135.0	18.92 [−10.26, 48.10]	0.2	96%
PLDRH vs. ODRH	11	2366	226.8 ± 94.4	216.4 ± 123.4	21.07 [−15.99, 58.12]	0.27	97%
LADRH vs. ODRH	7	684	264.3 ± 161.9	255.9 ± 163.7	9.67 [−26.24, 45.57]	0.6	56%
PSM	5	1060	226.1 ± 93.2	234.4 ± 93.0	−23.43 [−75.74, 28.89]	0.38	99%
**Peak TB**	17	3010	3.1 ± 1.6	3.1 ± 1.6	−0.08 [−0.26, 0.09]	0.36	83%
PLDRH vs. ODRH	11	2366	3.2 ± 1.6	3.0 ± 1.6	−0.04 [−0.25, 0.18]	0.73	88%
LADRH vs. ODRH	6	644	2.6 ± 1.4	3.2 ± 1.6	−0.21 [−0.42, −0.00]	0.05	0%
PSM	5	1060	3.3 ± 1.5	5.4 ± 12.2	0.08 [−0.38, 0.53]	0.74	96%
**Bile leak**	17	2958	37 (3.9%)	56 (2.8%)	1.28 [0.84, 1.97]	0.26	15%
PLDRH vs. ODRH	12	2346	29 (3.8%)	38 (2.4%)	1.46 [0.88, 2.46]	0.14	21%
LADRH vs. ODRH	5	612	8 (4.7%)	18 (4.1%)	0.90 [0.38, 2.11]	0.81	5%
PSM	5	823	12 (3.0%)	10 (2.3%)	1.32 [0.57, 3.08]	0.52	0%
**Clavien–Dindo grade ≥ III**	18	2904	47 (4.8%)	89 (4.6%)	1.06 [0.71, 2.24]	0.93	0%
PLDRH vs. ODRH	13	2260	36 (4.5%)	58 (3.9%)	1.07 [0.67, 1.72]	0.77	0%
LADRH vs. ODRH	6	644	11 (5.7%)	31 (6.9%)	1.04 [0.48, 2.24]	0.93	0%
PSM	6	1165	32 (5.7%)	29 (4.8%)	1.14 [0.68, 1.91]	0.63	0%
**Re-hospital**	7	1340	21 (6.5%)	39 (3.8%)	1.18 [0.68, 2.04]	0.56	0%
PLDRH vs. ODRH	5	902	14 (5.9%)	27 (4.1%)	1.16 [0.60, 2.25]	0.66	0%
LADRH vs. ODRH	2	438	7 (8.1%)	12 (3.4%)	1.21 [0.45, 3.25]	0.71	0%
PSM	3	332	12 (7.7%)	15 (8.5%)	0.87 [0.40, 1.93]	0.74	0%
**Reoperation**	13	2143	22 (3%)	27(1.92%)	1.43 [0.79, 2.57]	0.23	0%
PLDRH vs. ODRH	8	1531	15 (2.6%)	17 (1.7%)	1.47 [0.72, 2.99]	0.29	1%
LADRH vs. ODRH	5	612	7 (4.0%)	10 (2.3%)	1.33 [0.47, 3.78]	0.59	0%
PSM	3	322	5(3.1%)	3 (1.9%)	1.59 [0.41, 6.20]	0.50	0%
**Biliary stricture**	6	1467	8 (1.5%)	5 (0.5%)	2.38 [0.81, 7.04]	0.12	0%
PLDRH vs. ODRH	5	1317	7 (1.5%)	5 (0.5%)	2.18 [0.69, 6.90]	0.19	1%
LADRH vs. ODRH	1	150	1 (1.6%)	0 (0.0%)	4.56 [0.18, 113.89]	0.36	0%

^#^: in each group; CI: confidence interval; MIDRH: minimally invasive donors right hepatectomy; ODRH: open donors right hepatectomy; PLDRH: pure laparoscopic living donor right hepatectomy; LADRH: laparoscopic-assisted living donor right hepatectomy; Peak ALT: peak alanine aminotransferase; Peak AST: peak aspartate aminotransferase; Peak TB: peak total bilirubin.

## Data Availability

The original contributions presented in the study are included in the article. Further inquiries can be directed to the corresponding author.
